# Insight into a Fenton-like Reaction Using Nanodiamond Based Relaxometry

**DOI:** 10.3390/nano12142422

**Published:** 2022-07-15

**Authors:** Sandeep Kumar Padamati, Thea Annie Vedelaar, Felipe Perona Martínez, Anggrek Citra Nusantara, Romana Schirhagl

**Affiliations:** University Medical Center Groningen, University of Groningen, Antonius Deusinglaan 1, 9713AW Groningen, The Netherlands; sandeepimschcu@gmail.com (S.K.P.); solar_sola@hotmail.com (T.A.V.); felipeperona@gmail.com (F.P.M.); anggrek.citra.n@gmail.com (A.C.N.)

**Keywords:** NV-centers, nanodiamonds, copper, Fenton-like reactions, diamonds

## Abstract

Copper has several biological functions, but also some toxicity, as it can act as a catalyst for oxidative damage to tissues. This is especially relevant in the presence of H_2_O_2_, a by-product of oxygen metabolism. In this study, the reactions of copper with H_2_O_2_ have been investigated with spectroscopic techniques. These results were complemented by a new quantum sensing technique (relaxometry), which allows nanoscale magnetic resonance measurements at room temperature, and at nanomolar concentrations. For this purpose, we used fluorescent nanodiamonds (FNDs) containing ensembles of specific defects called nitrogen-vacancy (NV) centers. More specifically, we performed so-called T1 measurements. We use this method to provide real-time measurements of copper during a Fenton-like reaction. Unlike with other chemical fluorescent probes, we can determine both the increase and decrease in copper formed in real time.

## 1. Introduction

Copper is one of the vital elements present in cells. It is especially important in enzymes, which catalyze a variety of biological processes including oxidation, photosynthesis or cell wall metabolism [1]. However, copper in its free hydrated form, i.e., Cu^2+^, can be toxic to both plants and animals by altering membrane permeability and affecting chromatin structure, protein synthesis, and various enzyme activities [2]. In humans, several neurodegenerative diseases including Alzheimer’s and Parkinson’s disease are characterized by modified copper homeostasis [3]. Changes in copper metabolism in the brain either directly or indirectly increase oxidative stress, which is an important factor in neuronal toxicity. Copper-based materials are regarded as efficient catalysts in Fenton-like reactions at neutral pH and have thus been considered excellent candidates for developing new cancer treatments [4]. Notably, copper is used in chemodynamic therapy treatment for cancers by triggering ROS production. For this application, copper(I)-nanoparticles selectively kill tumor cells [5]. Another important process that involves copper in biology is the generation of hydroxyl radicals (HO●) and/or other reactive oxygen species (ROS). These are generated as a result of the reaction of copper with hydrogen peroxide (H_2_O_2_) in cells as a by-product of oxygen metabolism [6]. These reactions are studied by quantifying hydroxyl radicals using coumarin dyes [7] or other hydroxyl-specific dyes such as disodium terephthalic acid [8]. While these dyes have the advantage to be radical specific, they are limited by photo-bleaching and do not provide real-time measurements. Standard electron paramagnetic resonance (EPR) measurements can be used to detect copper(II). However, aqueous solutions are affected by the absorption of microwaves by water, which complicates measurements in biological samples [9]. In order to investigate Fenton-like reactions, in this case a reaction of copper with H_2_O_2_, we use T1-relaxometry in this article. This approach allows us to optically follow the reaction of copper with H_2_O_2_ at nanomolar concentrations in aqueous conditions, avoiding the effect of microwave absorption which is a bottleneck in conventional EPR.

So far, diamond magnetometry has been used for nanoscale magnetic resonance measurements and successfully applied to measure the magnetic field from nanoparticles [10], 2D materials, domain walls in magnetic structures [11], gadolinium ions in solution [12], spin-labeled molecules, proteins with metallic parts [13] or even slices of cells [14]. Recently, the first measurements in living cells have been demonstrated [15,16,17,18,19]. Here, we use a specific form of diamond magnetometry called T1 or relaxometry measurements [20]. This technique is suitable to measure paramagnetic copper(II) as well as spin noise from free radicals [21]. Recently, we used T1 magnetometry to detect the radicals formed during a Fenton reaction. More specifically, we investigated the hydrolysis of H_2_O_2_ during UV-irradiation and during the reaction of iron (II)perchlorate with H_2_O_2_ [12]. However, since copper is often used in chemotherapeutic studies and is preferred there over iron, we investigated the effect of copper and its reaction with H_2_O_2_ in this article. The concentrations we used are comparable with concentrations which appear in cells [22]. This is the first use of relaxometry for the measurement of copper generated by a copper-H_2_O_2_ reaction. Furthermore, we also complement these measurements with standard spectroscopic tools.

## 2. Materials and Methods

### 2.1. Materials

FNDs were purchased from ADAMAS nano, (Raleigh, NC, USA) average size ~70 nm as stock solution of 1 mg/mL. Each of these particles contains an ensemble of several hundred NV centers. These particles are produced from HPHT synthesis followed by milling and irradiation by the manufacturer. In the last step of the particle synthesis the particles are treated with oxidizing acid and thus are oxygen terminated. They are used widely for other applications and have been characterized extensively [23,24,25]. CuSO_4_ was purchased from the Alfa Aesar (Lancashire, United Kingdom). H_2_O_2_ (30 wt%), disodium terephthalate (Na_2_TH) and hydroxy terephthalic acid (HTA) was purchased from Sigma-Aldrich (St. Louis, MO, United States) and used without further purification. Petri dishes with glass bottom were purchased from Ibidi (Gräfelfing, Germany). All experiments were conducted at around ~22 °C, atmospheric pressure in aqueous solutions. Caution! The drying or concentration of solutions that potentially contain H_2_O_2_ should be avoided. Prior to drying or concentrating, the presence of H_2_O_2_ should be tested for using peroxide test strips followed by neutralization on solid NaHSO_3_ or another suitable reducing agent. When working with H_2_O_2_, suitable protective safeguards should be in place at all times due to the risk of explosion.

### 2.2. Relaxometry

The nanodiamond stock solution was diluted to 10 µg/mL using ultrapure water. An amount of 20 µL of this solution was added to a Petri dish with a glass bottom, which was pre-treated with air plasma for 15 min. After adding the solution, the Petri dish was kept in a fume hood for 1 h to evaporate the solvent. Then, 100 µL of ultrapure water was added to the plate and the first set of T1 measurements was performed. Further, solutions of CuSO_4_ were added until the respective concentrations (100 nM, 10 μM, 100 μM, 500 μM, 1 mM and 10 mM) were reached and T1 was measured. Another set of T1 relaxometry measurements was conducted with the same particle after adding H_2_O_2_ (30%) in the amount required to reach the specified concentration (10 nM, 100 nM, 1 mM, 10 mM). 

The diamonds for T1 measurements were selected based on intensity counts (between 10^6^ and 10^7^ counts per second) recorded by a home-built magnetometer. After appropriate particles were identified, the T1 was measured. This was performed by repeating the pulsing sequence shown previously in our group [12]. Obvious aggregates were excluded. During analysis, particles with a T1 higher than 600 µs were excluded. These values were likely caused by dirt or an outlier in the nanodiamonds (extremely large or small particles or fitting errors). First, the NV-centers in the nanodiamond were polarized by the laser (532 nm, Ventum, laser quantum, Novanta Photonics (Wackersdorf, Germany)). To ensure proper polarization, the nanodiamonds were illuminated for 5 µs and the dark time between pulses varied between 0.2 µs and 10 ms. The pulsing was applied by sending the pulsing scheme with a pulseblaster (Pulseblaster ESR pro, SpinCore) to an acousto-optical modulator (AOM, Gooch & Housego, Gainesville, Florida, United States). While a single experiment lasts microseconds, each experiment is repeated 10,000 times to improve the signal-to-noise ratio. The resulting experimental time is approximately 10 min. The photoluminescent signal was detected with a sensitive avalanche photo diode (APD). A long-pass filter (550 nm) was placed before the APD to only allow the fluorescence from NV centers to pass through. 

The fluorescence from the NV-centers was quantified in a detection window. This detection window was determined for each particle. The signal for each pulse was integrated over this window to obtain the T1 curve. This curve was fitted with a bi-exponential model (Equation (1)) as shown previously [12].

(1) PL(τ)=Iinf+Cae−τTa+Cbe−τTb

**Equation (1):** Equation for obtaining T1.

This model approximates that an ensemble of NV centers consists of 2 groups: one with a shorter T1 and one with a longer one. The latter was found to be most concentration dependent in earlier experiments.

Instead of using the entire 10,000 data points to generate one T1 value, it is also possible to increase the time resolution by using a moving window method. To this end the 1st–2500th repetitions are combined in one data point, the points 100–2600 form the second point and so on. This equals to approximately 2.5 min of experimental time. We repeat the process until the last repetition of a window ends at the 10,000th repetition.

The T1 values obtained during this process are not independent, due to the overlap between the different windows. Outliers in the moving window results were determined using the interquartile range (IQR).

### 2.3. CuSO_4_ Calibration Curve Using T1

A stock solution of CuSO_4_ (1M) was prepared by dissolving CuSO_4_ (2.49 g) in 10 mL of ultrapure water. For the calibration experiments, 100 µL of solvent (ultrapure water was taken in a petri dish with FNDs attached to it, and to this 1 M stock solution of CuSO_4_ solution was added gradually in increments to obtain the desired concentrations (0.1 µM, 10 µM, 100 µM, 500 µM and 1 mM). T1 measurements were recorded at each concentration according to the procedure described in Section 2.1. pH values for the solutions are shown in Supplementary Scheme S2.

### 2.4. UV–Vis Absorption Spectroscopy Measurements

UV/vis absorption spectra were recorded with a Specord600 spectrometer (AnalytikJena, Jena, Germany) in 10 mm path length quartz cuvettes. The spectroscopy was performed at room temperature from 180–1000 nm. UV–Vis absorption spectra were recorded at different time intervals The data analysis was performed by the Spekwin software (Spectragryph version 1.2.14. Author name: Dr. Friedrich Menges, Germany).

### 2.5. Measuring the Concentration of Hydroxyl Radicals by HTA

Disodium terephthalate (Na_2_TH) acts as chemical trap for hydroxyl radicals and is a standard hydroxyl dosimeter [8].

A calibration curve has been established with different concentrations of HTA against intensity using a fluorimeter (Edinburgh instruments (module sc-20), λ_Excition_ = 330 nm and λ_Emission_ = 420 nm). We performed a typical Fenton-like reaction using CuSO_4_ (1 mM), H_2_O_2_ (10 mM) and Na_2_TH (100 mM) in a quartz cuvette (10 mm pathlength). The solution (1.5 mL) contained CuSO_4_ (1 mM) and Na_2_TH (100 mM). Then, H_2_O_2_ (10 mM) was slowly added, and spectra were recorded at different time intervals. The fluorescence intensity was plotted for different concentrations of HTA to establish a calibration curve. From the calibration curve’s slope, we determined the concentration of hydroxyl radicals formed during the reaction of CuSO_4_ with H_2_O_2_.

### 2.6. Raman Spectroscopy

Raman spectra at 785 nm (300 mW at source, Cobolt Lasers, Hübner Photonics, Hannover, Germany) were acquired in a 180° backscattering arrangement. Raman scattering was collected by a 2.5 cm diameter plano-convex lens (f = 7.5 cm). The collimated Raman scattering passed through an appropriate long-pass edge filter (Semrock, Rochester, NY, USA) and was focused by a second 2.5 cm diameter plano-convex lens (f = 15 cm) into a Shamrock500i spectrograph (Andor Technology, Belfast, United Kingdom). We used a 2399 L/mm grating blazed at 300 nm and the data was collected with an iDus-420-BU2 charge-coupled device (CCD) camera (Andor Technology, Belfast, United Kingdom). The spectral slit width was set to 12 μm. Data were recorded and processed using Solis (Andor Technology, Belfast, United Kingdom) software. Spectral calibration was performed using the Raman spectrum of acetonitrile/toluene 50:50 (*v*:*v*). Samples were held in quartz cuvettes with a 10 mm path length. Multi point baseline correction was performed for all spectra.

### 2.7. Oxygen Sensor Measurements

A typical measurement was performed by taking CuSO_4_ (1 mM) 5 mL volume in a glass vial (15 mL). Then, H_2_O_2_ (10 mM) was slowly added. Oxygen sensor measurements were recorded using a WTW 2BA301 Oxi 3310. CuSO_4_ (1 mM) and H_2_O_2_ (10 mM) solutions were used for these measurements. The temperature for the measurements was around ~22 °C.

## 3. Results and Discussion

**Figure 1:**
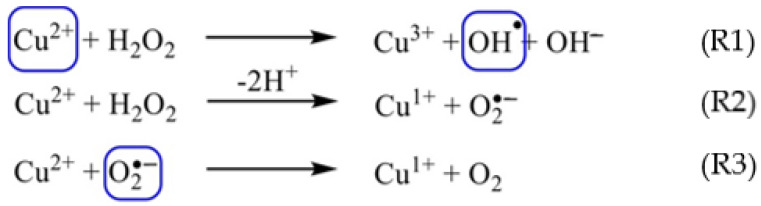
Reaction of copper(II) with H_2_O_2_ to form OH^●^ radicals and O_2_^●−^ (species that are paramagnetic are marked in blue).

**Figure 2:**
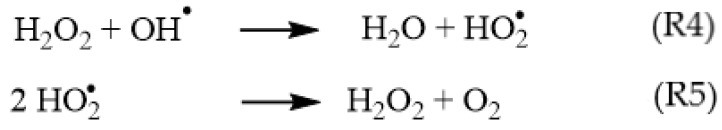
Radical formation from H_2_O_2_ and OH●.

We study the reaction of CuSO_4_ at low concentrations (<1 mM) with H_2_O_2_ (<10 mM) at room temperature using fluorescent nanodiamonds (FNDs) that contain NV centers. These NV centers detect paramagnetic species within about 20 nm range [26]. Through this method we follow the real-time changes in copper(II) concentration. The reaction of the copper(II) leads to the formation of hydroxyl radicals, and hydroxide ions (Figure 1 and Reaction (1)) and oxidized copper(III). The hydroxyl radicals have a chance to either react with another hydroxyl radical or they can react with hydrogen peroxide to form further hydroperoxide species. This in turn can lead to the formation of water and oxygen (see Reactions (4) and (5) in Figure 2). Another possible reaction path is the formation of a superoxide radical and copper(I). These superoxide radicals can react with excess copper in the solution to form Cu(I) liberating oxygen. As this reaction includes the formation of several radicals and copper oxidation states, it is a fast dynamic process. A technique that can provide a concentration of these species in real time at low concentrations is highly desirable.

### 3.1. T1-Relaxation Measurements: 

T1-relaxation measurements reveal the concentration of paramagnetic species present around the NV centers. In this paper, this is primarily copper(II). Superoxide radicals and OH● radicals which also occur in the reaction are present in relatively low concentrations and are short-lived and thus only detected in the higher concentrations. T1 measurements, which have been described before were performed as follows [12,27]. First, the laser brings the NV centers to the bright m_s_ = 0 state of the ground state. Then, we measure again after specific times (see the materials and methods sections for details) to see whether the NV centers are still in this state or not. Since the states differ in brightness (the m_s_ = 0 state is brighter) we can observe the process by recording the change in fluorescence. When there are flipping spins (in this case from copper or free radicals) in the surrounding, the NV centers will lose this state faster. Thus, the time that is required to lose the prepared state gives a quantitative measure for the concentration of these species. 

**Figure 3:**
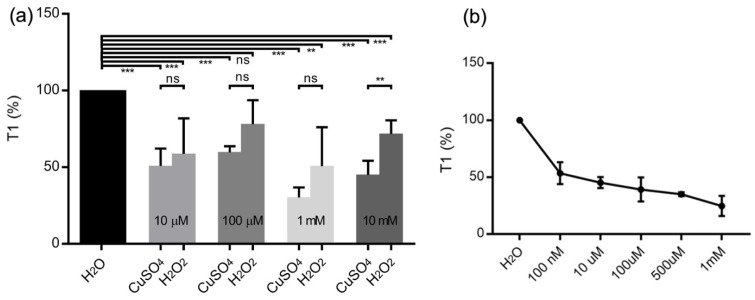
(**a**) T1—relaxation measurements of water, CuSO_4_ and after addition of H_2_O_2_ at different concentrations. Each bar represents an average of 4–5 independent measurements. (**b**) T1—relaxation measurements of CuSO_4_ at different concentrations (*n* = 4). The error bars represent standard deviations. (ns = non significant, * *p* ≤ 0.05, ** *p* ≤ 0.01, *** *p* ≤ 0.001, **** *p* ≤ 0.0001).

From Figure 3a, we see that the T1 relaxation times are normalized for ultrapure water for different concentrations of copper. After the addition of copper(II), the T1 value is decreased by more than 50% w.r.t ultrapure water, and further addition of H_2_O_2_ resulted in an increase in the T1 value. This shows the conversion of copper(II) to copper(I) or copper(III) as shown in the scheme above, which has resulted in an increase in T1. The data also supports the fact that copper(II) reacts with H_2_O_2_ to liberate OH● radicals (which were identified with other spectroscopic tools in the sections below) and the decrease in copper(II) concentrations over time. 

When lower concentrations of copper(II) were used (10 µM), we observe a smaller decrease in T1 value (Figure 3a) compared to higher concentrations of copper(II) in agreement with the copper calibration shown Figure 3b. A calibration curve of T1 values measured for copper(II) solutions in Figure 3b shows that the detection limit of the copper(II) with FNDs can reach nanomolar concentrations, confirming that copper(II) still can be detected in the solution in the reaction time scale (which is 20 min).

The most plausible reasons for the smaller decrease in T1 at lower concentrations of copper(II) 10 µM compared to 1 mM are mainly (a) the reaction of copper(II) of H_2_O_2_ takes place at a slower speed (around 20 min) compared to higher concentration (>1 mM), which leads to slower decrease in T1; (b) the concentration of H_2_O_2_ is 100 µM which is 100 times less than H_2_O_2_ used from higher concentration (10 mM). Therefore, the copper(II) decay is slow, because of the lower availability of H_2_O_2_. In this case, less H_2_O_2_ is present in relation to the copper(II) decay by different amounts of H_2_O_2_ present in solution. A similar situation has been described by the Pham et al. [28] (c) In addition OH● radicals produced in the solution at higher concentrations of H_2_O_2_ might be quenched quickly by reducing excess H_2_O_2_. In this process, they form peroxide radicals and further dimerize to form H_2_O_2_ and O_2_ as shown in Reactions 4 and 5. A similar observation has been described by Sapihu and co-workers, which is the most plausible steps in the OH● radicals decay process [29].

Another important point to consider is the adsorption of copper ions on the diamond surface. Since FNDs are electronegative, they can potentially interact with copper ions. This process has been described before in detail for detonation nanodiamonds [30]. To test this hypothesis, we conducted T1 measurements of two concentrations and then reversed the order of measuring. Indeed, we observed that after measuring a large concentration, we were not able to detect the small concentration accurately (See Supplementary Scheme S1). This is a common problem in analytical chemistry where it is common to measure small concentrations first or rinse thoroughly between measurements. 

### 3.2. CuSO_4_ Decay by UV–Vis Absorption Spectroscopy

**Figure 4:**
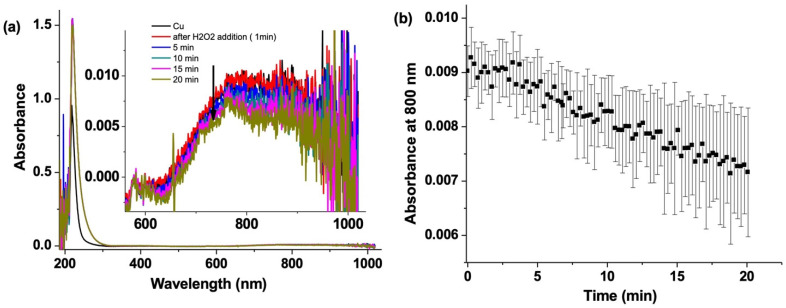
(**a**) UV–Vis absorption spectra of CuSO_4_ (1 mM) (black) and after addition of H_2_O_2_ and (**b**) its kinetic trace plotted at 800 nm over 20 min.

UV–vis absorption spectra of CuSO_4_ show a broad saturated band at 220 nm and a weak absorption at 800 nm, which is useful for monitoring the changes in the copper(II) concentration [31]. This band is caused by d-d band transitions, which indicate the concentration of copper(II) [32]. Figure 4 shows a clear decrease in absorption from copper(II) upon addition of H_2_O_2_. There is a change of 0.2 mM in concentration of copper over 20 min (based on the ε = 12.6 M^−1^ cm^−1^) [31]. This confirms that there is a reaction between copper(II) and H_2_O_2_. Pham et al. have shown that copper(II) reacts with H_2_O_2_ to form OH● radicals [28]. Thus, this is an example of a classical Fenton-like reaction. The studies here are consistent with the T1 relaxation measurements from Figure 3b, where there is an increase in T1 due to the decrease in copper(II) concentration over the reaction time.

### 3.3. Hydroxyl Radical Detection by Emission Spectroscopy

The fluorescent dye Na_2_TH was used to detect OH● radicals produced by the reaction of copper(II) with H_2_O_2_ (Figure 1 and Figure 2).

**Figure 5:**
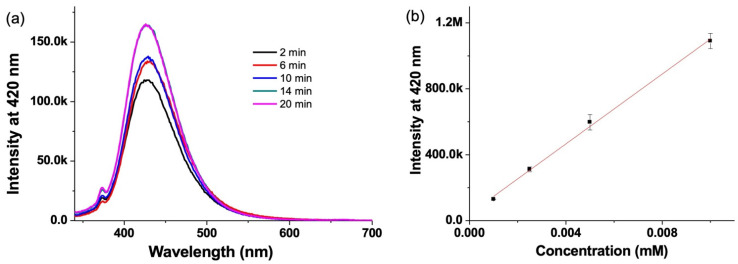
(**a**) Fluorescence spectra of HTA obtained by the reaction of CuSO_4_ (1 mM) and H_2_O_2_ (10 mM) with Na_2_TH (100 mM), 2 min (black), 6 min (red), 10 min (blue), 14 min (green) and 20 min (pink)) and (**b**) calibration curve obtained by plotting the fluorescence intensity at 420 nm for different concentrations of HTA.

Na_2_TH reacts specifically with hydroxyl radicals resulting in the formation of the fluorescent molecule hydroxyl tereprethalic acid (HTA). HTA is probed by excitation at 330 nm collecting the emission spectra at 420 nm (Figure 5a). In order to estimate the concentration of OH● radicals, a calibration curve of known concentrations of HTA was plotted. From this calibration, the concentration of OH● radicals produced in the reaction of CuSO4 with H_2_O_2_ was estimated to be 0.9 µM (Figure 5b).

### 3.4. H_2_O_2_ Decay by Raman Spectroscopy

**Figure 6:**
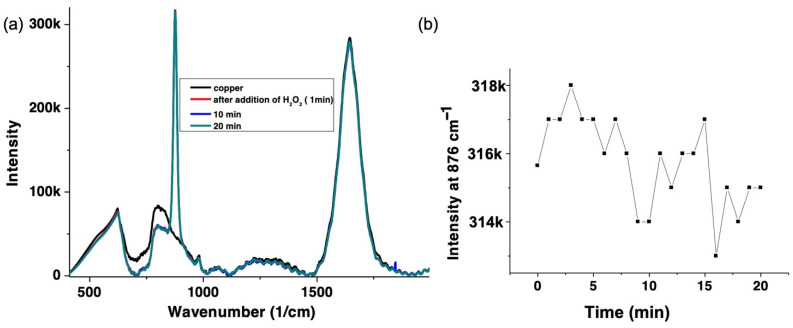
(**a**) Raman spectra of CuSO_4_ (1 mM) (black) and after addition of H_2_O_2_ (100 mM). Data points are shown at 1 min (red), 10 min (blue) and 20 min (green). (**b**) shows the intensity of the 876 cm^−1^ peak (H_2_O_2_ band (O-O band)) over 20 min detected by Raman spectroscopy with excitation at 785 nm. This indicates that the concentration of H_2_O_2_ is reduced during the reaction as outlined in Figure 1.

To quantify the decrease in the H_2_O_2_ concentration over time, Raman spectroscopy was used (Figure 6a). The O-O symmetric stretch, which is the signature band of H_2_O_2_ at 876 cm^−1^ and its intensity were followed over time at 785 nm [33]. Since there is no resonance enhancement of this band at 785 nm, we have used higher concentrations of H_2_O_2_, i.e., 100 mM H_2_O_2_. The 876 cm-1 band decreases by 3% over time (Figure 6b), which shows that roughly 0.3 mM of H_2_O_2_ has been consumed over the reaction time (20 min). This indicates that H_2_O_2_ reacts with copper(II), and is consistent with UV–vis absorption, fluorescence, and T1 measurements described above.

### 3.5. Oxygen Sensor Measurements:

**Figure 7:**
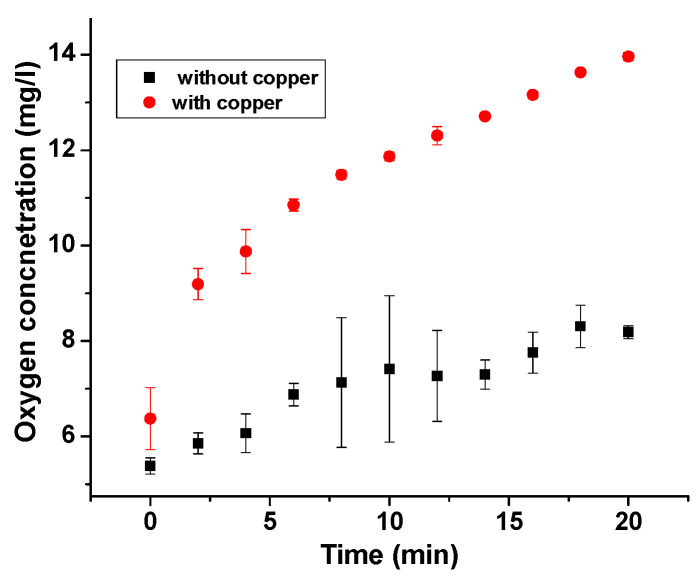
Dissolved oxygen measurements during the reaction of CuSO_4_ (1 mM) and H_2_O_2_ (10 mM) (red) and H2O_2_ added to ultrapure water (black).

Pham et al. proposed that oxygen is liberated from the reaction of copper(II) with H_2_O_2_ [28]. To confirm this hypothesis, we performed oxygen sensor measurements before addition of H_2_O_2_ to copper(II) (5 mg/L). Over the reaction time, there is a steady increase in the concentration of dissolved oxygen which suggests a clear liberation of O_2_ (14 mg/L) during the copper(II) reaction with H_2_O_2_ as shown in Figure 7.

### 3.6. Combined Measurements from Spectroscopic Techniques

**Figure 8:**
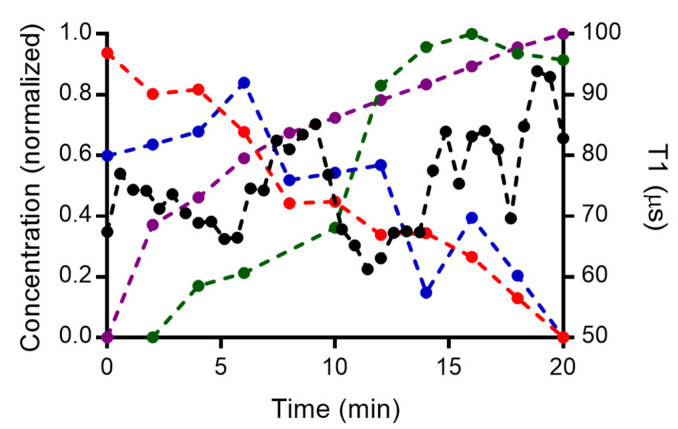
Normalized concentration changes of the different species obtained in the reaction of CuSO_4_ and H_2_O_2_. The changes T1 (black, averages from 9 particles are shown), the absorbance of copper (red), and H_2_O_2_ decay changes from Raman intensity (blue), fluorescence of the HTA for hydroxyl radicals (green) and the dissolved oxygen during the reaction (purple).

Figure 8 shows the normalized concentration of copper(II), H_2_O_2_, OH● radicals and oxygen liberated during the reaction of CuSO_4_ with H_2_O_2_ as well as our T1 measurements processed by the rolling window approach. We show the decrease in the concentration of copper(II) and H_2_O_2_, and an increase in hydroxide radicals and oxygen. These findings are very consistent with Figure 1. Hydroxyl radicals are produced in the solution and we observe a decrease in the copper(II) concentration which is consistent with the UV–vis absorption shown in Figure 4a. We also compare T1 obtained via the rolling window method with the conventional methods. We observed an initial decrease in water (100% in Figure 8) due to the presence of paramagnetic copper(II). As the reaction proceeds, the radicals are degraded and a decrease in copper(II) concentration occurs. T1 increases as a result. However, the T1 is not fully recovered to the level of the blank (only water), since there is a formation of hydroxyl radicals over time and not all copper(II) is consumed in the reaction.

### 3.7. Copper Detection by Existing Techniques

There are some other advanced spectroscopic techniques which are used to measure the copper concentrations as shown in Table 1 below. However, new technologies are emerging to study copper in lower concentrations in real time as well. These techniques are atomic absorption [34,35], emission [36] or mass spectrometry [37]. These are particularly useful to detect elements with high sensitivity. However, the ionizing or flaming of the solutes is a drawback and the methods do not provide a real-time analysis. Some label-free sensors, for instance photoluminescent polymer nanodots (PPNDs), were recently reported to detect copper(II). Liu et al. described the usage of these PPNDs to detect copper(II) by using fluorescence quenching down to 1 nM [38]. Another photoluminescence method to detect copper is using ligation to the copper(II) center. The 3-hydroxy-5-nitrobenzaldehyde-4-hydroxybenzoylhydrazone (3-HNHBH) ligand was used by Abdulazeez et al. to ligate to copper(II). This ligand is highly selective towards copper(II) ions and the authors were able to detect copper with a detection limit of 0.34 µgL^−1^ [39]. NV centers are infinitely stable and follow the paramagnetic species in real time at sub micromolar concentrations. We have shown copper(II) depletion in aqueous solutions using relaxometry. Additionally, magnetic resonance, in the long run, would allow us to differentiate between species using more complex pulsing schemes as the double electron resonance (DEER) can be used in near future [40].

## 4. Conclusions

In this work, we demonstrated the use of relaxometry measurements on a Fenton-like reaction (copper(II) and H_2_O_2_) and observed the decrease and increase in the concentration of paramagnetic species. We are able to measure nanomolar to micromolar concentrations of copper that are currently present, using low amounts of solution. Most importantly we can measure sub nanomolar concentrations of copper that are usually hard to detect in aqueous environments with standard EPR or ultraviolet–visible (UV–vis) absorption, because of the limitations in their detection range. We worked at concentrations that can be present in biological environments, which in the future can be applied to study the Fenton reactions in biological environments. It has to be noted that in this specific case (next to copper) we are not able to detect the hydroxyl radicals themselves. The observation of copper(II) would help in other biological fields such as targeting cancer cells with copper nanoparticles. Relaxometry can be potentially applied to study the killing mechanism in tumor cells. The tools applied here would be a great asset in the future to study reactions in cells, which are catalyzed by copper systems such as copper nanoparticles. However, one has to be cautious if large concentrations are measured before small ones due to an adsorption of copper ions on the FND surface.

## Figures and Tables

**Table 1:** Comparing sensing performance of different techniques.

Technique	Concentration	Pros and Cons	Mechanism and Information Obtained	Reference
FAAS (flame atomic absorption spectrometry)	47–1888 nM	-destructive -spatial resolution +relatively simple	Atomic absorption on the sample is recorded. It is an invasive technique, but is highly sensitive to the element of choice.	[33]
ETAAS (Electrothermal atomic absorption spectrometry)	8 nM	-destructive -spatial resolution +high sensitivity		[34]
ICP OES (Inductively coupled plasma optical emission spectrometry)	19 nM	-destructive -destructive +relatively simple +high sensitivity	The mass spectra were recorded by emission spectroscopy. It is an invasive technique, and highly selective to copper.	[35]
ICP-MS (inductively coupled plasma mass spectrometry)	1 nM	-Expensive equipment -destructive +high sensitivity	The solution mass spectra were recorded, the method is highly specific to the particular element and highly sensitive to copper.	[36]
Cu(II)-DNAzyme	100 nM	+Non-destructive +spatial resolution	Cu(II) binds to DNA, which leads to the release of fluorophore (6-carboxyfluorescein). Hence a fluorescence signal from the fluorophore is detected.	[41]
FNDs (fluorescent nanodiamonds)	100 nM	-specialised equipment +Non-destructive +spatial resolution	Change in T1 time in presence of paramagnetic species. Copper(II) can be quantified down to nanomolar concentrations.	This work

## Data Availability

All data in relation to this manuscript are available in the paper or on request from the authors.

## Supplementary Materials

The following supporting information can be downloaded at: https://www.mdpi.com/article/10.3390/nano12142422/s1, Scheme S1. Testing copper adsorbtion on FNDs. To test if copper adsorbs to the FNDs we reversed the order of experiments; Scheme S2. pH values of the differently concentrated copper sulfate solutions with and without FNDs.
