# Supplementary material for: Insight into a Fenton-like Reaction Using Nanodiamond Based Relaxometry
Source: Nanomaterials (Basel). 2022 Jul 15;12(14):2422. doi: 10.3390/nano12142422 (PMC9319944; doi:10.3390/nano12142422)
Supplement: Supplementary file 1 [file nanomaterials-12-02422-s001.zip › nanomaterials-1720855-supplementary.pdf]

*nanomaterials*

*Supplementary Material*

# Insight into a Fenton-Like Reaction Using Nanodiamond Based Relaxometry

**Sandeep Kumar Padamati †, Thea Annie Vedelaar †, Felipe Perona Martínez, Anggrek Citra Nusantara and Romana Schirhagl ***

University Medical Center Groningen, University of Groningen, Antonius Deusinglaan 1, 9713AW Groningen, The Netherlands; sandeepimschcu@gmail.com (S.K.P.); solar_sola@hotmail.com (T.A.V.); felipeperona@gmail.com (F.P.M.); anggrek.citra.n@gmail.com (A.C.N.)

* Correspondence: romana.schirhagl@gmail.com

† These authors contributed equally to this work.

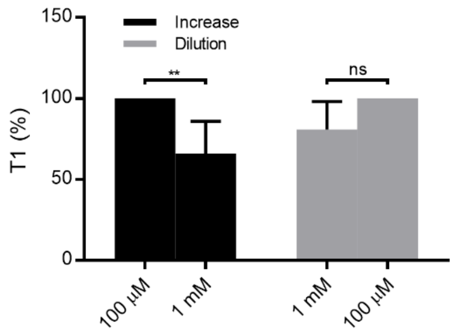


**Scheme S1.** Testing copper adsorbtion on FNDs. To test if copper adsorbs to the FNDs we reversed the order of experiments.

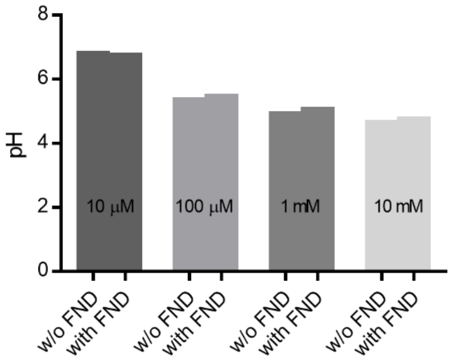


**Scheme S2.** pH values of the differently concentrated copper sulfate solutions with and without FNDs.
